# Burden of neurological and neurocognitive impairment in pediatric sickle cell anemia in Uganda (BRAIN SAFE): a cross-sectional study

**DOI:** 10.1186/s12887-019-1758-2

**Published:** 2019-10-25

**Authors:** Nancy S. Green, Deogratias Munube, Paul Bangirana, Linda Rosset Buluma, Bridget Kebirungi, Robert Opoka, Ezekiel Mupere, Philip Kasirye, Sarah Kiguli, Annet Birabwa, Michael S. Kawooya, Samson K. Lubowa, Rogers Sekibira, Edwards Kayongo, Heather Hume, Mitchell Elkind, Weixin Peng, Gen Li, Caterina Rosano, Philip LaRussa, Frank J. Minja, Amelia Boehme, Richard Idro

**Affiliations:** 1 Department of Pediatrics, Columbia University Vagelos Medical Center, 630 West 168 St., Black Building 2-241, Box 168, New York, NY USA; 2 Department of Paediatrics and Child Health, Makerere University College of Health Sciences, Kampala, Uganda; 3 Department of Psychiatry, Makerere University College of Health Sciences, Kampala, Uganda; 4 Department Radiology, Makerere University College of Health Sciences, Kampala, Uganda; 5 Department of Paediatrics, CHU Sainte-Justine, University of Montreal, Montreal, Canada; 6 Departments of Neurology, Epidemiology and Biostatistics, Columbia University Vagelos Medical Center, New York, NY USA; 7 Department of Biostatistics, Mailman School of Public Health, Columbia University Vagelos Medical Center, New York, NY USA; 8 Epidemiology and of Clinical and Translation Science, University of Pittsburgh, Pittsburgh, PA USA; 9 Department of Pediatrics, Columbia University Vagelos Medical Center, New York, NY USA; 10 Department of Radiology, Yale University, New Haven, CT USA; 11 Department of Neurology, Columbia University Vagelos Medical Center, New York, NY USA

**Keywords:** Sickle cell anemia, Stroke, Neurocognitive impairment, Transcranial doppler, Sub-Saharan Africa

## Abstract

**Background:**

Children with sickle cell anemia (SCA) are highly susceptible to stroke and other manifestations of pediatric cerebral vasculopathy. Detailed evaluations in sub-Saharan Africa are limited.

**Methods:**

We aimed to establish the frequency and types of pediatric brain injury in a cross-sectional study at a large SCA clinic in Kampala, Uganda in a randomly selected sample of 265 patients with HbSS ages 1–12 years. Brain injury was defined as one or more abnormality on standardized testing: neurocognitive impairment using an age-appropriate test battery, prior stroke by examination or transcranial Doppler (TCD) velocities associated with stroke risk in children with SCA (cerebral arterial time averaged mean maximum velocity ≥ 170 cm/second).

**Results:**

Mean age was 5.5 ± 2.9 years; 52.3% were male. Mean hemoglobin was 7.3 ± 1.01 g/dl; 76.4% had hemoglobin < 8.0 g/dl. Using established international standards, 14.7% were malnourished, and was more common in children ages 5–12. Overall, 57 (21.5%) subjects had one to three abnormal primary testing. Neurocognitive dysfunction was found in 27, while prior stroke was detected in 15 (5.7%). The most frequent abnormality was elevated TCD velocity 43 (18.1%), of which five (2.1%) were in the highest velocity range of abnormal. Only impaired neurocognitive dysfunction increased with age (OR 1.44, 95%CI 1.23–1.68), *p* < 0.001). In univariate models, malnutrition defined as wasting (weight-for-height ≤ −2SD), but not sex or hemoglobin, was modestly related to elevated TCD (OR 1.37, 95%CI 1.01–1.86, *p* = 0.04). In adjusted models, neurocognitive dysfunction was strongly related to prior stroke (OR 6.88, 95%CI 1.95–24.3, *p* = .003) and to abnormal TCD (OR 4.37, 95%CI 1.30, *p* = 0.02). In a subset of 81 subjects who were enriched for other abnormal results, magnetic resonance imaging and angiography (MRI/MRA) detected infarcts and/or arterial stenosis in 52%. Thirteen subjects (25%) with abnormal imaging had no other abnormalities detected.

**Conclusions:**

The high frequency of neurocognitive impairment or other abnormal results describes a large burden of pediatric SCA brain disease in Uganda. Evaluation by any single modality would have underestimated the impact of SCA. Testing the impact of hydroxyurea or other available disease-modifying interventions for reducing or preventing SCA brain effects is warranted.

## Background

Approximately 225,000 children with sickle cell anemia (SCA) are born in sub-Saharan Africa each year [1]. In Uganda, 15,000–20,000 affected infants are born annually, most with homozygous SCA (HbSS) [2].

In affected children, SCA brain vasculopathy may cause overt stroke, primarily ischemic in nature, starting early in childhood. Without preventative measures, children with HbSS are 200-fold more susceptible to stroke compared to the general pediatric population [3]. Prior to implementation of prevention strategies, frequency of childhood stroke in the U.S. and Jamaica was 5–10%, with first stroke in early childhood [3–5]. Vasculopathy of subcortical or small vessels in SCA may also cause silent cerebral infarction (SCI), lacking an associated neurologic event. At times, one or more SCI may manifest as neurocognitive dysfunction [6–12].

The large number of children with SCA in sub-Saharan Africa, coupled with high frequency of stroke risks such as severe anemia and limited available medical resources contribute to a high burden of sickle cerebral vasculopathy and associated SCA brain injury [13–15]. Targeted stroke prevention employs risk assessment by transcranial doppler (TCD) to detect abnormal flow in large cerebral arteries, but is rarely available in the region [6]. Systematic multi-modal evaluations of structural or functional impact of pediatric sickle cerebral vasculopathy in sub-Saharan Africa have been limited [16–24]. Previous brain imaging studies by computed tomography or magnetic resonance (MRI) have been rare and small in scope [25].

We aimed to determine the frequency and characteristics of neurocognitive and neurological abnormalities of young children in a randomly selected sample from a large SCA clinic in Kampala for the study “Burden and Risk of Neurological and Neurocognitive Impairment in Pediatric Sickle Cell Anemia in Uganda (BRAIN SAFE).”

## Methods

### Study design

BRAIN SAFE was a cross-sectional study of children with confirmed HbSS (or HbSB^0^ thalassemia) receiving care from the SCA clinic at Mulago Hospital in Kampala, Uganda. Approximately 3500 patients actively receive care at this clinic and are included in the clinic’s electronic clinical database [26]. Study documents used for caretakers and subjects were written in English and translated into Luganda, a predominant local language, by a certified translator, then reviewed by the study team for accuracy.

### Eligibility, recruitment, screening and enrollment

Study inclusion criteria: ages 1–12 years, prior clinical laboratory documentation of SCA (HbSS or HbS-B^0^ thalassemia) by standard hemoglobin electrophoresis, prior SCA clinic appointments (or one appointment for age ≤ 2), hemoglobin (Hb) ≥6.0 g per deciliter (g/dl) (HemoCue AB, Sweden). Additional inclusion criteria were absence of an acute crisis, acute illness with fever or respiratory infection over the preceding 2 weeks per parental report, or blood transfusion during the preceding 3 months.

#### Exclusion criteria

history of neurological impairment prior to age 4 months to help avoid subjects with non-SCA neurological events (e.g. complicated birth, neonatal brain injury), study participation of a sibling, past or concurrent enrollment in a clinical intervention study or history of hydroxyurea use.

Sample size was determined by age trends for SCI detected by magnetic resonance imaging (MRI) in U.S. and French children with SCA. SCI is the most common type of early brain vascular injury detected by MRI, is associated with cognitive dysfunction, and generally is not prevented by TCD screening [6]. Based on the estimated frequency of SCA cerebral vasculopathy of 20% within the study age range from elsewhere, [6] 80% power within a 95% confidence interval, the target sample size was 246. To account for an estimated 8% incomplete testing due to attrition, logistics or death, study entry was offered to 265 sequentially eligible participants.

### Subject selection and assessment

**Fig. 1:**
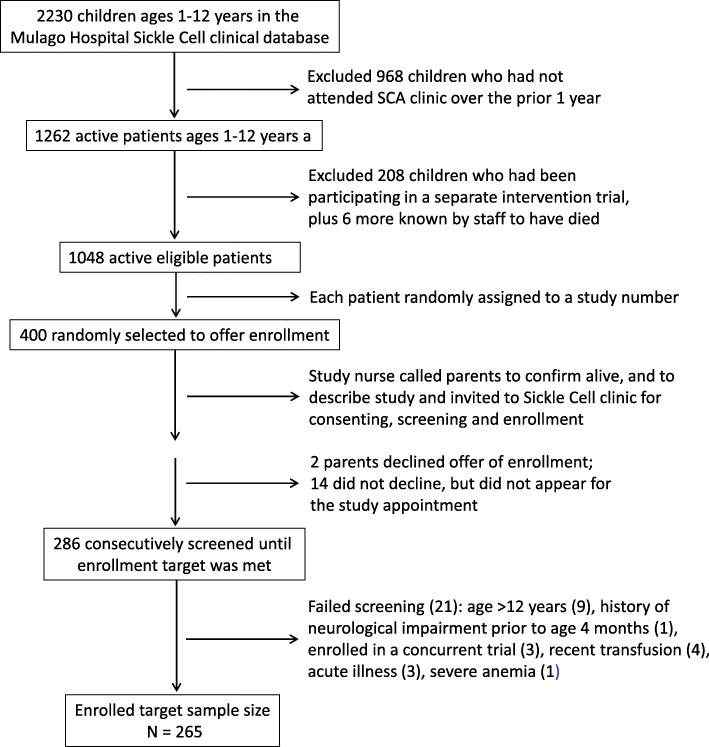
Flow chart for subject recruitment and enrollment

From the clinic’s electronic clinical database, patients with correct study ages and who had attended clinic at least once in the prior 12 months were randomly assigned a study number from the electronic SCA clinic roster at Mulago Hospital (Fig. 1), where 2230 children with SCA were registered in the clinical database [27]. Study numbers were randomly assigned to the 1048 active patients ages 1–12 years. Among those, parents or guardians of a random sample of 400 were sequentially telephoned by the study nurse to mention the study and invite the child to the clinic for screening. This process was repeated until the target enrollment number was achieved. Patients meeting all criteria were offered enrollment. Written parental consent and (if applicable) assent (for children 8 years or older) were obtained.

Demographic (sex, age) and clinical (Hb, *anthropometry*) variables were collected at enrollment, as was a detailed SCA history. Stroke examination and TCD measurement were also performed on the day of enrollment. Other testing, described below, were performed by appointment at other study visits. Detailed patient histories for prior strokes or other complications and hospitalizations, had not been routinely recorded in the clinical database or other medical source document. Hence medical history was not included in this report.

#### Anthropometric assessment

Study staff determined routine height or length and weight measurements according to World Health Organization (WHO) standard procedures for growth monitoring of delayed or inadequate growth [28]. “Using established international pediatric standards, the WHO defines “wasting,” a type of malnutrition, as weight-for-height/length z-scores or BMI of ≤ − 2SD, by age and sex, for children younger or older than age 5 years, respectively. Severe wasting is defined as z-scores of ≤ − 3SD [28].

In children 2 years or older, the standing (upright) height was determined using a portable stadiometer. For children < 2 years or those unable to stand upright, recumbent length was measured. Weight was determined using a calibrated digital scale. These anthropometric measures were selected to reduce influence from disease-associated delayed growth and development that are frequently found in pediatric SCA across resource settings [16, 29].

### Hematological assessment

Following enrollment, a complete blood count (CBC) was obtained and assayed by the hospital’s central laboratory using standard parameters.

### Neurocognitive and neurologic results

Subjects underwent three different standardized neurologic or neurocognitive assessments to detect abnormal findings in one or more assessment. A selected subset underwent a fourth assessment, described below.

### Neurocognitive testing

A standardized age-appropriate test battery was administered to each subject on a separately scheduled day by testers who were experienced in the materials used. Test materials had been previously translated into and validated in Luganda [30–34]. Testing and scoring were performed by trained, experienced testers who were closely supervised by a senior study team member. Testing was performed at a separate study visit to avoid participant fatigue. Subjects ages 1–4 years underwent testing using the Mullen Scales of Early Learning, testing for gross and fine motor skills, expressive and receptive language, and visual reception [35, 36]. Subjects aged 5–12 years were tested using the Kaufman Assessment Battery for Children, 2nd edition (KABC-II), testing for short- and long-term memory, reasoning, ability and visual processing [37, 38]. In each test, summation of subscales generated an overall score of neurocognitive ability. Abnormal results were defined as ≤ − 2 z-scores below age-specific established community norms, based on previously determined data for children without SCA within the same age range: 106 controls for the KABC-II and 149 for the Mullen [30, 33].

### Stroke assessment

The standardized well-validated pediatric NIH Stroke Scale, PedNIHSS, was used for documenting prior stroke in children ages 2–18 years. PedNIHSS scores are assigned to define the presence and degree of stroke findings: 0 (no stroke), 1–4 (mild stroke), 5–15 (moderate stroke), 16–20 (moderately severe stroke) or 21–42 (severe stroke) [39, 40]. Testing was performed by a study staff physician following training and initial direct supervision by the study pediatric neurologist. Parental report was not used to identify subjects with prior stroke, as subject histories had not been medically documented. While seizures can be associated with SCD stroke in the West, [41] seizures from endemic CNS infections are more common in sub-Saharan Africa [42].

### TCD velocity

Non-imaging TCD standardized for pediatric SCA was performed on subjects ages ≥2 years by two study staff using a single machine (SonaraTek, Natus, U.S.). Subjects undergoing testing were afebrile, awake, and did not have a recent acute illness. Arterial time-averaged maximum mean velocities (TAMV) were performed bilaterally over the middle (MCA), anterior and posterior cerebral (PCA) and internal carotid arteries (ICA) [43]. Increased stroke risk with elevated flow velocities in one or more arteries tested, defined by standardized criteria as elevated: conditional (≥170 - < 200 cm/second) or abnormal (≥200 cm/second), or very low, predicts high SCA stroke risk in children ages 2–16 years [43, 44]. Very low TAMV (< 70 cm/s), excluding the PCA and distal ICA, is also a risk factor for stroke [46]. TCD was considered to be inadequate if readings did not include a minimum of TAMV for bilateral MCA and internal carotid arteries [47].

### Training and tester reliability

Training, supervision and quality assurance were performed [43]. The intra-class correlation coefficient statistic was used to assess reliability between the two testers following their training and supervision, per Galadanci et al. [45]

(See Additional file 1.)

### TCD protocol

Per standardized approach, subjects with initial abnormal or conditional velocities underwent repeated testing within 1–3 months [43, 47]. Upon re-testing, subjects with persistent abnormal or TCD velocities were referred to SCA clinic for initiating hydroxyurea therapy, per the clinic’s standardized protocol for dosing and monitoring [27].

### Brain MRI/MRA imaging

Non-contrast imaging was performed on a 1.5 T scanner (1.5 T Achieva MRI equipment from Philips Medical Systems, Netherlands). Based on available study budget, a 30% subset of subjects (*N* = 81) underwent MR imaging. To investigate the correlation between imaging markers and abnormal testing, this subset was intentionally over-sampled for having abnormal test results in at least one of the three primary evaluations. The subset included 61 participants with abnormal testing, with 20–25 each with abnormal TCD or cognitive impairment, and 14 of the 15 with prior stroke, plus 20 who had normal test results in all three domains. Selection for MR imaging was made irrespective of age or sex.

Scans were performed on medically stable subjects. Oral sedation by chloral hydrate was used if needed for agitation. Standard clinical imaging interpretation was performed by two study radiologists who were blinded to other study results. Size of infarct and arterial stenosis were determined for pediatric SCA [46, 48].

### Data management

An electronic study database was developed by and housed on a secure server using EPI-DATA version 3.1 software package (The EpiData Association, Odense, Denmark) by Global Health Uganda.

### Statistical analyses

Primary abnormal results were a documentation of prior stroke on the PedNIHSS stroke exam, an impaired neuro-cognitive test result on the Mullen’s or KABC II test compared to historical community controls or TAMV by TCD outside of the normal range for pediatric SCA (conditional or abnormal), reported as proportions. Relationships between the sample characteristics and the results were examined by ANOVA or chi square tests, as appropriate. A 2-tailed probability test was used, with statistically significant *P*-values defined by <.05. Logistic regression was used for multivariate analyses. Each model was run with the maximum sample size possible when some covariates were not collected. Analyses were conducted using IBM SPSS Statistics (version 25, NY). Standardized mortality ratio was used to estimate rates of mortality and stroke over the brief observational period [49].

## Results

### Sample description

Of 400 randomly selected patients eligible by diagnosis and age, study enrollment was offered until reaching the enrollment target of 265 (Fig. 1). Two parents declined enrollment outright, while 14 did not attend the study appointment and declined further telephone calls from study staff. Of 286 patients screened, 265 were eligible (92.3%), and all of these enrolled. Ineligibility was due to age > 12 years (9), recent transfusion (4), current acute illness (3), screened Hb < 6.0 (1), history of early neurologic impairment (1), participation in another concurrent study (3), or unrecorded reason (1). Among enrolled participants, mean age was 5.5 ± 2.9 years (Table 1); over half were pre-school. Children ages 1–4 and 5–8 years each constituted 40.7% of the sample, while subjects ages 9–12 years were 18.6%. The sample was 52.5% male.

**Table 1:** Sample characteristics of 265 subjects with SCA, ages 1–12 years

	Total Mean ± SD *N* = 265 (%)	Ages 1–4 years Mean value ±SD *N* = 113 (%)	Ages 5–12 years Mean value ±SD *N* = 152 (%)
Age (years)	5.5 ± 2.9	2.5 ± 1.1	7.5 ± 2.1
Sex (male), N (%)	139 (52.4)	61 (54.0)	77 (50.6)
Height (cm) (*N* = 252)	109.3 ± 18.0	92.0 ± 13.7	121.6 ± 11.9
Weight (kg) (N = 252)	18.1 ± 6.1	13.8 ± 9.6	22.7 ± 12.6
Malnutrition^+^ (N = 252), N (%)	37 (14.7)	10 (9.3)	27 (18.8)
Severe malnutrition^++^, N (%)	12 (4.8)	3 (2.8)	9 (6.3)
Malnutrition (z-score)	−0.82 ± 1.52		
Weight-for-height (z-score)	–	−0.49 ± 1.75	–
BMI z-score	–	–	−1.06 ± 1.27
Hemoglobin^#^ (g/dl) (*N* = 250)	7.3 ± 1.0	–	–
Hb ≤7.5 g/dl (*N* = 158)	6.7 ± 0.5	6.6 ± 0.6	6.7 ± 0.5
Hb > 7.5 g/dl (*N* = 92)	8.3 ± 0.7	8.4 ± 0.7	8.3 ± 0.8

^+^Defined per World Health Organization standards, by age and sex, for children 1–4 years and ages 5–12 years, as z-score of ≤ − 2; ^++^z-score of ≤ − 3. (ref. 28)

^#^Severe anemia is defined as Hb < 8.0 g/deciliter (National Cancer Institute CTCAE Version 5.0, 2017)

Over half of the participants in the BRAIN SAFE cross-sectional sample were pre-school age. Participants were divided into two age groups to align with the neurocognitive testing batteries used for these two age ranges. Malnutrition (low weight-for-height) was highly prevalent, especially among those ages 5–12 years. Hemoglobin levels were low and did not vary by age

### Hematological and anthropometric assessment

Mean Hb was 7.3 ± 1.01 g/dl. (Table 1), with 76.4% having severe anemia, defined as < 8 g/dl (National Cancer Institute CTCAE Version 5.0, 2017). Although all participants were screened by Hemacue for Hb ≥6.0 g/dl, lower Hb was detected by CBC in 19 (7.6%). Of them, 18 had Hb 5.0–5.9 g/dl, one had 4.6 g/dl. No subjects had reported acute symptoms of anemia.

Using WHO definitions and standards by age and sex, 37 of 252 participants with adequate measurement (14.7%) were malnourished, with z-scores of ≤ − 2 (Table 1). Older subjects ages 5–12 years were more affected by malnutrition: 27 of 144 (18.8%). Among these, nine (6.3%) were severely malnourished, with z-scores of ≤ − 3. In contrast, among subjects ages 1–4, 10 of 108 (9.3%) were malnourished; three (2.8%) were severely malnourished.

### Neurocognitive and neurological results

Overall, 57 (21.5%) subjects had one or more abnormal results detected (Table 2). The subset with abnormal MRI/MRA despite normal testing added an additional 5.7% detected pathology.

**Table 2:** Proportion of subjects affected by abnormal results in one or more of the three primary tests performed, overall and by age group

	Entire Cohort Mean value (N = 265)	Ages 1–4 years Mean value N = 113 (%)	Ages 5–12 years Mean value N = 152 (%)
Any of the 3 tests performed	57 (21.5%)	22 (23.7%)	43 (31.2%)
Mean age for any abnormality (SD)	6.17 (3.3)	2.45 (0.74)	8.07 (2.32)
Prior stroke (N = 265)	15 (5.7%)	3 (3%)	12 (8.5%)
Mean age for prior stroke (SD)	6.4 (2.56)	2.33 (0.58)	7.42 (1.62)
Elevated TCD (*N* = 251)	43 (17.1%)	17 (18.9%)	18 (13.6%)
Abnormal TCD (*N* = 5)	5 (2.0%)	1 (1%)	3 (2%)
Conditional TCD (*N* = 38)	38 (15.1%)	20 (20%)	18 (12.3%)
Mean age for elevated TCD (SD)	4.92 (3.18)	2.38 (0.74)	7.48 (2.56)
Neurocognitive Dysfunction (*N* = 246)	27 (11.0%)	5 (5%)	22 (15.5%)
Mean age for neurocognitive dysfunction (SD)	8.04 (3.12)	2.60 (0.89)	9.27 (1.80)

One in five participants (57 of 265, 21.5%) had at least one abnormal result in the three tests performed. The most frequent findings were elevated TCD velocity and neurocognitive dysfunction

### Neurocognitive testing

The parents of 21 participants refused to return for a separate study appointment; their children missed testing. Using age-specific locally standardized test scoring, 27 of 244 subjects tested (11.1%) had abnormal subscale summary scores: 3 of 100 (3.0%) ages 1–4 and 25 of 144 (16.7%) ages 5–12. (Table 2).

### Stroke assessment

Standardized PedNIHSS [40] revealed that 15 of 262 (5.7%) had an abnormal neurological examination consistent with prior stroke. (Table 2) Nine subjects had unilateral stroke findings in the minor range (scale 1–4), and six within the moderate range (5–15). The two highest stroke scores were 14 and 15, which fall within the highest levels within the moderate range [39]. Age of subjects with stroke findings did not differ from age distribution of the entire sample (Table 2). For this study, stroke findings were gauged as prior stroke or not.

### TCD: tester reliability

Volunteers for testing operator reliability were 40 subjects ages 2–12 years and 10 young adults without SCA. The MCA right and left velocities measured by each examiner were normally distributed. Testers met criteria for high reliability for each MCA side tested, expressed by Cronbach’s alpha was ≥0.85 for each side tested [50].

### TCD examination

We attempted to perform TCD testing on all subjects. Three were unable to schedule a TCD examination. Of 262 participants with attempted TCD testing, bilateral readings were obtained in 251. Of the 11 participants whose TCDs were unable to be performed despite two separate testing sessions, nine had poor bone windows on one or both sides (“inadequate”) one was uncooperative and one was younger than age 2 years.

Among 251 subjects with successful bilateral TAMV measurements, bilateral TCD velocities ranged from 93 to 250 cm/sec. Based on standardized velocities for pediatric SCD, [43] five subjects (2.1%) had abnormal measures: 212–250 cm/sec. An additional 38 (16.0%) had conditional results of 170–191 cm/sec (Table 2), a total of 43 (18.1%) with elevated velocities (Table 2). No participant had very low flow velocities. Age was not a risk factor for elevated velocity (Table 3, Fig. 1). Forty of 43 participants with elevated TCD returned within 1–3 months for repeat testing. Of the five subjects with an initial abnormal velocity, four remained in the abnormal range and one became conditional. Overall, 30 participants had velocities still above the limit for normal pediatric SCD [43]. Parents of these children agreed to the clinic’s standard treatment regimen of hydroxyurea.

**Table 3:** Uni- and multi-variate analyses for study variables assessed for each of the three primary tests performed: neurocognitive dysfunction, abnormal TCD and prior stroke^a^

	Unadjusted			Adjusted*		
	OR	95%CI	*p*-value	OR	95%CI	
Neurocognitive Dysfunction (N = 246)						
Age (years)	1.44	1.23–1.68	**< 0.0001**	1.50	1.26–1.79	**< 0.0001**
Sex (male)	1.01	0.45–2.24	0.98	–	–	–
Hemoglobin (grams/dl)	1.09	0.74–1.62	0.65	–	–	–
Malnutrition (z-score)^+^	0.80	0.62–1.04	0.09	0.83	0.59–1.17	0.29
Abnormal TCD^++^	2.82	1.06–7.50	**0.04**	–	–	–
Prior stroke	6.67	2.16–20.6	**0.001**	6.88	1.95–24.3	**0.003**
Abnormal TCD (N = 252)						
Age	0.92	0.81–1.04	0.19	0.90	0.77–1.05	0.18
Sex	0.90	0.45–1.82	0.77	–	–	–
Hemoglobin (grams/dl)	0.93	0.64–1.34	0.69	–	–	–
Malnutrition	1.37	1.01–1.86	**0.04**	1.36	0.99–1.85	**0.05**
Neurocognitive Dysfunction	2.82	1.06–7.50	**0.04**	4.37	1.30–14.7	**0.02**
Prior stroke	0.69	0.08–5.72	0.74	–	–	–
Prior stroke (*N* = 264)						
Age	1.12	0.94–1.34	0.20	1.01	0.84–1.23	0.86
Female Sex	0.96	0.34–2.74	0.94	–	–	–
Hemoglobin (grams/dl)	1.19	0.73–1.95	0.48	–	–	–
Malnutrition^+^	0.81	0.58–1.13	0.21	–	–	–
Abnormal TCD	0.69	0.08–5.72	0.73	–	–	–
Neurocognitive Dysfunction	6.67	2.16–20.6	**0.0010**	6.27	1.79–22.0	**0.004**

*For each outcome, adjusted models were constructed using each of risk factors collected (sex, age, hemoglobin and malnutrition) and other outcome(s) identified as associated in the univariable analyses. Hemoglobin and age were kept as continuous variables in the model

^+^Defined per World Health Organization standards, by age and sex, for children 1–4 years and ages 5–12 years, as z-score of ≤ − 2 (ref. 28)

^++^TCD data refer to abnormal velocities, including those in the “conditional” and “abnormal” ranges for pediatric SCA (ref. 43)

Individual and potentially overlapping associated risk factors collected were assessed separately for each of the three abnormal primary test results. Only neurocognitive dysfunction was significantly related to age and to abnormal results in each of the other tests. Malnutrition was associated with elevated TCD and showed a trend to neurocognitive dysfunction, while hemoglobin and age were not associated with any of the three primary results. Overall, the most significant predictor of poor outcome was other poor outcomes, holding age and malnutrition constant. Prior stroke was most strongly associated neurocognitive dysfunction. In contrast, prior stroke was not associated elevated TCD

### MRI/MRA imaging

A subset of 81 with MR imaging was enriched for MR imaging was enriched for those with one more abnormal primary test result, mean age 6.5 ± 2.76 years, 41 (50.1%) male. In all, 42 had an abnormal scan (51.9%). The ages of children with normal compared to abnormal MR imaging was not significantly different: age 6.0 ± 2.99 versus 6.8 ± 2.24 years.

Among the abnormal scans, one or more medium or large infarcts were seen in 35 (83%), and multiple small infarcts in seven (17%) [46]. MRA detected ≥1 arterial stenosis in nine (21%); all but two were accompanied by one or more infarcts. Of the 20 participants intentionally sampled for having normal results on all three of the primary tests, 12 were found to have 1–2 small cortical or ≥ 1 subcortical infarcts. These subjects constituted an additional 4.5% of the overall sample with brain abnormalities detected. Statistical relationships between participants with abnormal imaging and each of the three primary other tests were not explored due to the non-random sampling.

### Descriptive and statistical analyses of primary results

Overall, 57 (21.5%) subjects had ≥1 abnormal primary test result(s), 17 (6.9%) had ≥2 abnormal primary results and 2 (1.0%) had all three abnormalities tested (Table 2). As expected by frequency, the most overlapping results were conditional TCD and neurocognitive impairment. Each continuous clinical variable (age, Hb, malnutrition) was normally distributed. In a correlation analysis malnutrition was strongly associated with Hb (*p* = 0.004) and older age (*p* < 0.001), but not with sex. Only neurocognitive dysfunction significantly increased with age (OR 1.44, 95%CI 1.23–1.68, p < 0.001) (Table 3). In adjusted models, prior stroke was related to a six-fold increased risk of neurocognitive dysfunction (*p* = .003), but not elevated TCD velocity. In addition, neurocognitive dysfunction was associated with elevated TCD velocity (*p* = 0.02), but not prior stroke (Table 3).

Stroke was strongly related to neurocognitive impairment in univariate and adjusted models, as was elevated TCD (*p* = 0.003; Table 3). Malnutrition was independently associated with elevated TCD (*p* < 0.04), but an association with neurocognitive impairment was not statistically significant. Other demographic (age, sex) and clinical variables (Hb and malnutrition) had no significantly association. Relationships with the results of MR imaging were not examined due to non-random selection of the subsample of subjects with imaging.

Mortality and incident stroke

Over a 5-month observational period during completion of study procedures, three subject deaths (1.1%) occurred. Of these deaths, two were attributable to acute stroke. By stroke examination, no prior stroke had been detected in either participant. One of the stroke deaths occurred in a child with an abnormal TCD, prior to return for repeat TCD testing and initiation of hydroxyurea. Etiology of the third death was a different SCA complication, acute chest syndrome. Based on these data, there were an estimated 1.8% strokes per year prorated from a 5-month observational period. No other strokes were known to have occurred during the study procedure period of this cross-sectional study by parental report or clinic-based follow-up.

## Discussion

This study, BRAIN SAFE, used multiple standardized neurological and neurocognitive analyses to estimate neurological and neurocognitive impairment or risk. Our main finding was the high frequency – overall one in five - of one or more brain abnormalities tested, or one in four if including SCI from the subset with MRI/MRA. Excluding the sampling for MR imaging, not many subjects had more than one abnormality detected.

Other key findings were: 1) only poor neurocognition depended on age, and rarely was found before age 5; 2) neurocognitive impairment was strongly associated with abnormally high TCD velocity, also reported in the U.S. and Nigeria, [52, 53], and with stroke (reviewed in ref. 6); 3) malnutrition was also a marker of stroke risk; 4) severe anemia, a major risk factor for impaired neurocognition, abnormal TCD and SCI in pediatric SCA [6, 17, 51, 54], was seen in three-quarters of our sample; 5) some participants with normal neurological and neurocognitive testing were found to have cerebrovascular abnormalities on MR imaging. These results collectively suggest that Ugandan children with SCA are severely affected by brain abnormalities, and exhibit potentially modifiable risk factors of malnutrition and anemia. Lack of an age-dependent accumulation of prior stroke or elevated TCD velocity may reflect a possibility that large vessel disease predisposes to increased clinic drop-out or mortality.

Recent SCA studies in children of comparable ages in other African countries have reported similar or higher frequency of abnormal TCD results [16, 17, 19, 20, 23].; some studies included neurocognitive assessments [20, 24]. Testing restricted to one modality, e.g. TCD, may underestimate the effect of SCA on brain injury in African children [16, 18, 55, 56]. We are aware of only one other African report which also includes SCA stroke examination and MR imaging [20] i.

The estimated mortality rate from deaths during our relatively brief observational period was comparable to that reported in a 36-month Nigerian study of children with SCA and similar age range [16]. Observed mortality in children of this age range is consistent with prior regional estimates for SCA [57, 58].

Our findings resemble U.S.-based natural history observations from a HbSS cohort: frequent severe SCA cerebral vasculopathy in early childhood, including abnormal MR imaging and neurocognitive function [59, 60]. Important differences compared to the Dallas cohort and others were the suggestion of high mortality and lack of statistical association with low Hb [61, 62]. These differences may reflect the impact from additional risk factors in the region: endemic infections, pervasive severe anemia and frequent malnutrition [63].

### Study limitations

Age of onset of the abnormalities detected could not be determined. Prior stroke may have been underestimated due to incomplete clinical documentation. Fnot been included. Distinction between HbSS and HbSB^0^ thalassemia was not evaluated; few of the latter would be expected in Uganda [2]. Subjects with very low Hb were unintentionally included due to inaccurate screening Hb. Severe anemia and malnutrition appear to be common among the SCA clinic’s pediatric patients. Two different measures of nutritional assessment, weight-for-height for ages 1–4 and BMI for ages 5–12, were used to take advantage of the more recent established data for age-specific international norms, and to align with the age ranges for the two neurocognitive testing batteries used. The newer data reflects improved rates of childhood mortality and health status seen over the past decade in Uganda and most of sub-Saharan Africa [64]. All three primary tests were not obtained for each participant due to logistic or technical rather than systematic challenges. The proportion of fetal hemoglobin (HbF%), an important disease modifier, was not obtained. In a sample of 216 children with SCA from the same clinic, mean age 9.3 ± 4.8 years, the mean HbF% was 9.0 ± 5.58 [65]. Alpha thalassemia trait also was not evaluated [19]. Other than the historic community controls for neurocognitive testing, local children lacking SCA were not assessed. Disease-related mortality may have skewed participants towards younger ages and survivors. Non-neurological factors contributing to abnormal neurocognitive testing were not assessed, e.g. socio-economic factors or parental education. MR imaging was not performed for all subjects, nor on a random sub-sample.

## Conclusions

High frequency of neurological and neurocognitive results were found in a clinic-based sample of young children with SCA. Pediatric SCA stroke in high-resource countries has markedly decreased, largely from primary prevention through TCD screening [43, 66, 67] as well as increased use of hydroxyurea and other preventative health measures [68, 69]. Primary stroke prevention through use of hydroxyurea rather than chronic blood transfusions in low-resource settings appears to reduce TCD velocities and stroke risk [16, 18, 55]. Recent Africa-based treatment trials in SCA indicate its feasibility, including in Uganda [27, 70]. Our findings, along with those cited herein, underscore the imperative testing the impact of hydroxyurea or other available disease-modifying interventions to reduce or prevent pediatric SCA brain vasculopathy in Africa.

## Supplementary information

**Additional file 1.** Transcranial doppler ultrasound: training and tester reliability.

## Data Availability

Study materials used are available from the corresponding author upon reasonable request.

## Abbreviations

CBC: Complete blood count
Hb: Hemoglobin
HbSB^0^ thalassemia: Compound heterozygous HbS and beta thalassemia 0
HbSS: Homozygous SCA
KABC-II: Kaufman Assessment Battery for Children, 2nd edition
MRI/MRA: Magnetic resonance imaging and angiography
PedNIHSS: Pediatric NIH Stroke Scale
SCA: Sickle cell anemia
TAMV: Time-averaged maximum mean velocities
TCD: Transcranial Doppler
WHO: World Health Organization
