# Supplementary material for: Burden of neurological and neurocognitive impairment in pediatric sickle cell anemia in Uganda (BRAIN SAFE): a cross-sectional study
Source: BMC Pediatr. 2019 Oct 25;19:381. doi: 10.1186/s12887-019-1758-2 (PMC6814102; doi:10.1186/s12887-019-1758-2)
Supplement: Supplementary file 1 — Additional file 1. Transcranial doppler ultrasound: training and tester reliability. [file 12887_2019_1758_MOESM1_ESM.docx]

**Additional file 1.**

**Transcranial doppler ultrasound: training and tester reliability**

**Methods:**

*TCD Training*: A company representative provided technical training for investigators on the study machine. One investigator had previously been trained and certified for TCD performance and interpretation in the U.S.[43] This investigator supervised both study staff who performing testing for the first 190 subjects and performed standard readings of all arterial velocities.[43] Training for the two study staff was initiated by a one-week intensive training program conducted by an research nurse from Augusta University with extensive experience on performing and teaching TCD for pediatric SCA.[45] Quality assurance for TCD tracings was performed by a stroke neurologist from Columbia University.

*TCD tester reliability*: The intra-class correlation coefficient statistic was used to assess reliability between the two testers following their training and supervision, per Galadanci et al.[46] Right and left MCA velocities were sequentially assessed on volunteers with or without SCA by each of the two testers within one hour of each other.
